# 30 years after the fall of the Berlin Wall: Regional health differences in Germany

**DOI:** 10.25646/6077

**Published:** 2019-11-01

**Authors:** Thomas Lampert, Stephan Müters, Benjamin Kuntz, Stefan Dahm, Enno Nowossadeck

**Affiliations:** Robert Koch Institute, Berlin Department of Epidemiology and Health Monitoring

**Keywords:** REGIONAL DIFFERENCES, EAST AND WEST GERMAN FEDERAL STATES, LIFE EXPECTANCY, HEALTH, RISK FACTORS

## Abstract

Following the fall of the Berlin Wall in November 1989, considerable effort was made to bring the living conditions and levels of social participation in the former East German federal states into line with the former West German federal states. As a result, differences in health between the East and the West diminished significantly, in many cases as early as the 1990s, examples being life expectancy and cardiovascular mortality. In regard to health behaviour, the overall tendency has also clearly been one of convergence. Thus, only very small differences can be observed today, for example in the use of tobacco or in the prevalence of obesity. Yet the results also highlight the insufficiency of regarding the remaining differences as a simple comparison between East and West. Instead, the focus should shift towards smaller-scale approaches that take regional differences in living conditions into account.

## 1. Introduction

After the fall of the Berlin Wall in November 1989, significant efforts were made to bring the living conditions and levels of social participation in the former East into line with the former West. Over the past 30 years, this has been achieved in many areas. Standard wages in East Germany, for example, are now 98% of the level in the West, and pensions in the East are set to achieve parity with pensions in the West by 2024 [1]. Differences in living standards between East and West, i.e. people’s access to those consumer goods and durables they consider important, as well as their subjective satisfaction with living conditions, have also reduced significantly [2]. On the other hand, unemployment rates and the proportion of people affected by or at risk from poverty continue to be significantly higher in the former East German federal states. However, this situation should not be ascribed to German Democratic Republic (GDR) history alone, but rather viewed against a backdrop of current economic, social and demographic developments.

It is clear that migration processes as an immanent element of social change vary significantly in the former East and West German federal states. Immigration from abroad has focused in particular on former West Germany. Moreover, many people have migrated and continue to migrate from East Germany to West Germany, which means that former East Germany has lost a significant number of people emigrating to former West Germany, predominantly young people and young, highly educated women (so-called age-selective migration) [3]. Thus, women leaving these declining regions either became or might potentially become mothers, which has led to a spiral of emigration and declining birth rates [4]. Although there are indications that an increased number of people are now returning to the East [5], the majority of those returning are men.

The 2009 report, ’20 Jahre nach dem Mauerfall: Wie hat sich die Gesundheit in Deutschland entwickelt?‘ strikingly demonstrates how important the living conditions and levels of social participation in former East and West Germany have been to people’s health [6]. Drawing on a broad data basis, this report from the Robert Koch Institute, which was commissioned by the Federal Ministry of Health, takes stock of developments in health within East and West Germany. It covers an array of topics, from disease and mortality to health behaviour and its associated risk factors, and healthcare. As in the follow-up article published five years later [7], the initial report highlights the fact that many of the differences between health outcomes for the East and West have either diminished or no longer exist. Such is the case for average life expectancy, subjective health, as well as numerous chronic diseases and underlying risk factors. Moreover, the report clearly shows the inadequacy of an approach that focuses on the remaining differences between East and West. Instead, a smaller-scale regional analysis should be aimed for, one which takes regional differences in living conditions into account, for example in regard to economic power as well as employment and income opportunities [6-8].

In the present article in the Journal of Health Monitoring, we continue from previous reports, concerning ourselves on the 30th anniversary of the fall of the Berlin Wall with the question of whether the described developments have continued, and if so, to what extent. Initially, the article examines average life expectancy and mortality (Chapter 3.1), before shifting focus to cardiovascular diseases, cancer and mental disorders (Chapter 3.2 to Chapter 3.4). In addition to subjective health (Chapter 3.5), tobacco use, obesity and physical inactivity (Chapters 3.6 to Chapter 3.8) are considered as important behavioural risk factors. To conclude, selected examples are used to illustrate the importance of a smaller-scale analysis of East-West differences that, where possible, takes the often substantial differences in people’s living conditions and opportunities for social participation into account (Chapter 3.9).

## 2. Methodology

The findings presented in this article draw on a broad data basis. There are, however, very few studies and other data sources available that allow comparisons between the health of people in the former GDR and those in former West Germany. The current analysis, therefore, begins after the German reunification in 1990, when substantial efforts were made to improve the data basis for such comparisons. Between 1991 and 1992, for example, the Robert Koch Institute conducted a health survey in former East Germany that was broadly based on the health survey conducted in former West Germany between 1990 and 1991 [9, 10]. The first national health survey, the German National Health Interview and Examination Survey 1998, was conducted between 1997 and 1999 [11]. The present report also analyses a number of Robert Koch Institute surveys such as the German Health Interview and Examination Survey for Adults (DEGS1, 2008-2011) [10], as well as several waves of the German Health Update (GEDA) that were conducted in 2009, 2010, 2012 and 2014/2015 [12]. Findings on cardiovascular diseases are grounded in data on the lifetime prevalence (whether a person has ever contracted the disease in the course of their life) of significant cardiovascular disease (self-reported medical diagnosis of stroke, heart failure, infarction or any other coronary heart disease). GEDA 2014/2015-EHIS surveyed the prevalence of current depressive symptoms via the Patient Health Questionnaire (PHQ-8) [13].

Analyses of incidence case figures (number of new cases) or incidence rates (number of new cases per 100,000 inhabitants) draws on data from the German Centre for Cancer Registry Data at the Robert Koch Institute. Besides data from the Robert Koch Institute, this report also uses data from the Socio-Economic Panel (SOEP) of the German Institute for Economic Research [14] and the Microcensus of the Federal Statistical Office [15, 16]. Moreover, key insights are provided by further data sources from official statistics such as vital statistics [17], causes of death [18] and hospital diagnoses [19]. These findings are supplemented by results from individual health studies and/or surveys such as the survey of the German Olympics Sports Federation on membership numbers at sports clubs [20].

To compare disease and mortality rates of population groups with diverse age structures, age standardisations were calculated based on the old European standard population [21]. To analyse and evaluate regional health differences on a smaller scale than that of the federal state, these differences should be considered alongside differences in living conditions and opportunities for social participation. The Robert Koch Institute has developed a corresponding index, which measures regional differences in socioeconomic deprivation (German Index of Socioeconomic Deprivation, GISD) based on a set of indicators that include unemployment, employment rate, net household income, debt ratio, tax revenue and the number of school leavers who leave without a certificate [22]. Multidimensional indices at the regional level are advantageous because they not only depict individual aspects but also the total socioeconomic advantages and disadvantages of specific socio-spatial areas.

## 3. Results

### 3.1 Life expectancy

Shortly after the German reunification, average life expectancy at birth was still significantly lower in the former East German states than in the West. Women in former East Germany had an average life expectancy at birth of 77.2 years, while in the West it was 79.5. For men, the difference was even greater, with 69.9 years compared to 73.1. In subsequent years, the East-West differences in average life expectancy at birth rapidly decreased. Parity for women was almost achieved in 2000, and since 2015/2017, the average life expectancy for women in former East Germany has even become slightly higher than in the former West German federal states [23, 24]. While male life expectancy has not achieved parity, the differences between the former East and West Germany have decreased significantly. As with women, this can be traced back to developments in the 1990s. The remaining disparity of slightly over a year less for men in former East Germany has not diminished any further (Table 1).

A corresponding development can also be observed in regard to life expectancy at age 65. At the beginning of the 1990s, life expectancy at age 65 was one to two years lower for women and men from the former East German federal states compared to women and men from the former West. These rates have now more or less equalised; the life expectancy of women at age 65 is now marginally higher in former East Germany, while 65-year-old men from former East Germany still have a slightly lower life expectancy at age 65 than men from the former West.

### 3.2 Cardiovascular diseases

The prevalence of cardiovascular diseases in former East Germany is higher than in former West Germany. GEDA data from 2009, 2010 and 2012 indicates that the lifetime prevalence of significant cardiovascular diseases (self-reported medical diagnosis of heart attack, stroke or heart failure) in the former East German federal states (including Berlin) was 13.0%, and therefore higher than in the former West German federal states (excluding Berlin) (11.7%) [25].

**Figure 1:**
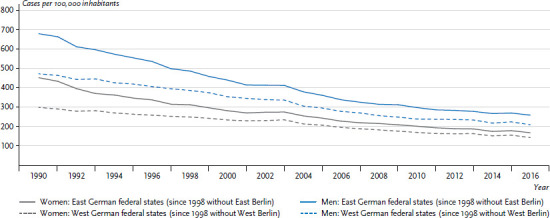
Development of cardiovascular mortality (age-standardised) in the former East and West German federal states 1990-2016 by sex Source: Cause of death statistics [18]

Mortality due to cardiovascular diseases is also elevated in the former East German federal states (Figure 1). While cardiovascular mortality rates had already begun to drop during the 1980s, convergence with levels encountered in the former West German federal states began after 1988 [24]. In 1990, age-standardised cardiovascular mortality in the former GDR was 1.52 times higher for women and 1.44 times higher for men compared to West Germany. This higher mortality then fell, and by 2016 it stood at 1.18 for women and 1.24 for men.

Eight largely avoidable risk factors (behavioural risk factors such as physical inactivity, drinking risky amounts of alcohol, smoking and low consumption of fruit and vegetables, as well as disease-related risk factors such as obesity, hypertension, diabetes and lipometabolic disorders) are behind over half of cardiovascular-related deaths [26]. The regional distribution of these risk factors in Germany was examined using data from the GEDA studies 2009, 2010 and 2012. Significant variations in prevalence among the federal states were found for all eight risk factors. Former East German federal states, with the exception of Berlin, presented the highest prevalence of physical inactivity, obesity, hypertension and diabetes for both sexes and risky alcohol consumption for men. The three city-states Berlin, Hamburg and Bremen ranked highly for smoking (Chapter 3.6). The prevalence of consuming less than one portion of fruit, vegetable or juice per day was greatest in Saarland [27].

### 3.3 Cancer

The German Centre for Cancer Registry Data at the Robert Koch Institute estimated cancer incidence (without non-melanocytic skin cancers) in 2014 to be around 227,000 cases for women and 249,000 for men. Among women in 2014, the highest incidences were recorded for breast cancer (30.5% of new cases), bowel cancer (12.3%) and lung cancer (8.5%). For men, the highest incidences were found for prostate cancer (23.0% of new cases), lung cancer (13.9%) and bowel cancer (13.3%) [28]. Between 1970 and 2014, the annual number of new cancer incidences doubled. Since the risk of developing most types of cancer increases with age, around half of this increase can be explained by demographic ageing, i.e. a shift in the age structure of the population towards a greater proportion of people in older age groups [29, 30]. Further factors, such as changes to behavioural risk factors, cancer-screening tests and improved diagnostic procedures, have also contributed to this increase [30].

**Figure 2:**
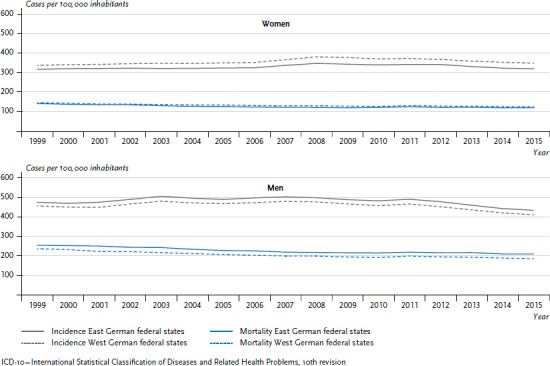
Development of cancer incidence and mortality (ICD-10: C00-C96, excluding C44) per 100,000 inhabitants (age-standardised) in the former East and West German federal states 1999-2015 by sex Source: German Centre for Cancer Registry Data [28]

Differences vary in overall cancer incidence rates (number of new cases (incidences) per 100,000 people) between the former East and West German states. Women in former East Germany present lower incidence rates than women in the West, while the opposite applies to men (Figure 2). At the same time, these differences have remained stable over the past years. Cancer mortality is dropping, and for women, there are only minimal differences between the former East and West German federal states (Figure 2). For men, the differences are greater, to the detriment of those in former East German states, and these differences have not diminished in the period discussed here.

**Figure 3:**
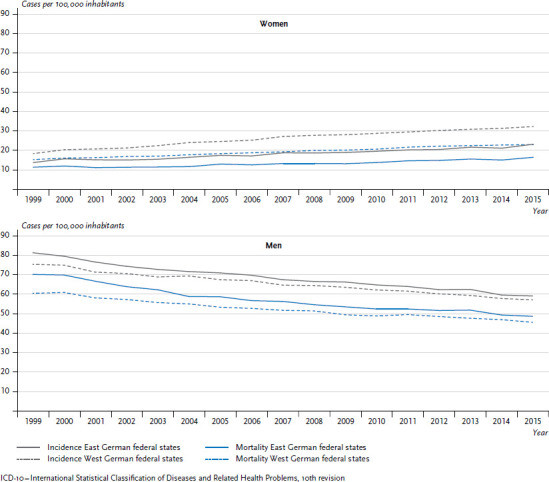
Development of lung cancer incidence and mortality (ICD-10: C33-C34) per 100,000 inhabitants (age-standardised) in the former East and West German federal states 1999-2015 by sex Source: German Centre for Cancer Registry Data [28]

A different picture emerges for lung cancer. Within the trend towards an overall increase, lung cancer incidence rates and mortality for women are lower in the former East German federal states than in the former West German federal states. For men, the situation is different. Incidence rates and mortality in former East Germany are higher than in the West, while the overall trend is towards a decrease (Figure 3). The differences between women in the former East and West German states have increased, following a sharper increase in mortality in the former West German federal states than in the East. This is mainly owing to the proportion of female smokers, which was higher in the West for many years (Chapter 3.6). Differences in smoking rates between East and West have only converged in recent years [7].

Since the consequences of smoking often only manifest themselves as lung cancer many years later, around half of patients develop the disease after the age of 70, i.e. decades after they took up smoking. For this reason, the present lowered smoking rates will only be reflected in lower incidence and mortality after another two to three decades [31].

**Figure 4:**
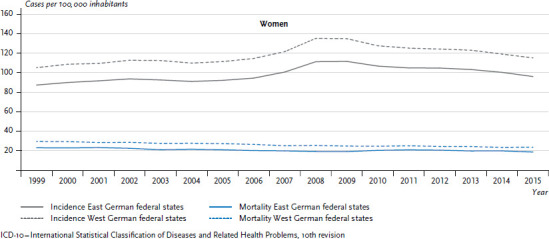
Development of breast cancer incidence and mortality (ICD-10: C50) per 100,000 inhabitants (age standardised) in the former East and West German federal states 1999-2015 Source: German Centre for Cancer Registry Data [28]

Breast cancer incidence rates in the former East and West German federal states continued to rise until 2008/2009, in particular after 2005. Since then, incidence rates have been decreasing (Figure 4) as a result of the mammography screening programme established between 2005 and 2009 [32]. For women aged 50 to 69, access to the programme is free of charge. The programme helps diagnose tumours at an earlier stage. The earlier diagnosis leads initially to a rise in incidence rates followed by a drop [30]. During the entire observation period, the incidence rate of breast cancer is markedly higher in the former West German federal states than in the former East federal states.

Breast cancer mortality has been falling in both the East and the West German federal states since the 1990s (Figure 4), although mortality rates in the West are still roughly 20% higher than in the East. Higher birth rates, the lower age of first-time mothers, fewer women who opt not to have children and other lifestyle factors are assumed to be protective factors for women in the East German states [30, 33-35].

### 3.4 Mental health

Mental disorders are widespread and come with a high burden of disease [36]. Analysis of the additional mental health module from the DEGS1 study (DEGS1-MH) by the Robert Koch Institute shows that the general 18- to 79-year-old German population has a 27.7% 12-month prevalence for mental disorders. At the same time, the differences between the East and West German federal states are rather small. Whereas 36.6% of women in the East have been diagnosed with some kind of mental disorder, the figure for women in the West is 33.7%. The prevalence in men in the former East German federal states is 20.4%, compared to 23.0% in the former West German federal states [7, 37].

According to GEDA 2014/2015-EHIS, the prevalence of current symptoms of depression in Germany is 10.1% [13]. It is slightly lower in East Germany (9.1%) compared to West Germany (10.3%). 10.8% of women from the East German federal states present with symptoms of depression, whereas for women in West Germany the figure is 11.7%. The prevalence in men is 7.3% and 8.9%, respectively. The 12-month prevalence of a self-reported medical diagnosis of depression in Germany, which GEDA 2014/15-EHIS also collected data on, is 8.1% [38], with East Germany at 6.6%, lower than West Germany at 8.3%. 8.8% of women from the East German federal states suffer from a medically diagnosed depression, compared to 9.8% in the West German federal states. Prevalence in men is 4.2% compared to 6.7%. Differentiated analyses by federal state show that both depressive symptomatology and self-reported medical diagnoses of depression are more frequent in the West German federal states. One exception is Bavaria, where the prevalence is lower. Prevalence is higher in city-states than in non-city federal states [13].

Similar distributive patterns between the East and West German federal states are also reflected in health insurance billing data on depressive disorders [13, 38, 39]. Thus, when considering different age and sex structures, depression is more frequently diagnosed in the West German federal states than in the East [39]. Besides true differences in disease prevalence, possible explanations under discussion include disparities between health-care systems. Diagnoses should, for example, be made by psychotherapists and psychiatrists. Particularly in the case of psychotherapists, there is a far greater concentration in the West German federal states than in the East, which means there are also better chances for diagnosis [39-41].

Hospital diagnosis statistics can provide further information on the treatment of mental disorders. A significant increase in the number of inpatient cases of mental and behavioural disorders was registered in the period from 2000 to 2011, a trend that has, however, not continued. This development can be seen in both the female and male population, and as much in the East as in the West German federal states. Since the turn of the century, the differences between the East and West German federal states have not further increased.

**Figure 5:**
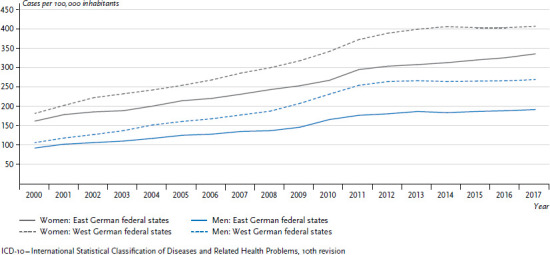
Development of inpatient case numbers for affective disorders (ICD-10: F30-F39) per 100,000 inhabitants (age-standardised) in the former East and West German federal states 2000-2017 by sex Source: Hospital diagnoses statistics [19]

**Figure 6:**
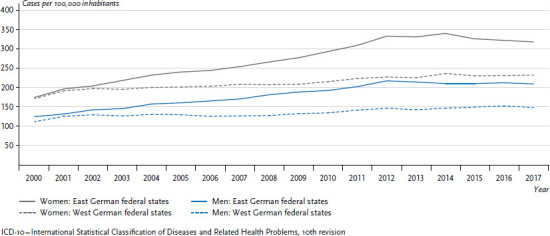
Development of inpatient case numbers for neurotic, stress-related and somatoform disorders (ICD-10: F40-F48) per 100,000 inhabitants (age-standardised) in the former East and West German federal states 2000-2017 by sex Source: Hospital diagnoses statistics [19]

It is a different story when considering specific groups of mental disorders. This is the case, for example, for the affective disorders (International Statistical Classification of Diseases and Related Health Problems, 10th revision, ICD-10: F30-F39), to which depression belongs, alongside neurotic disorders, stress disorders and somatoform disorders (ICD-10: F40-F48). Whereas inpatient case numbers in the diagnostic group of affective disorders are higher in the former West German federal states than in the East (Figure 5), these are higher in East Germany for neurotic disorders, stress-related disorders and somatoform disorders (Figure 6). These differences between East and West German federal states became greater between 2000 and 2012 but have not diverged any further since.

### 3.5 Subjective health

**Figure 7:**
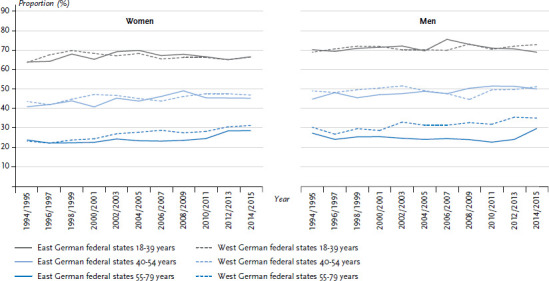
Prevalence of ‘good’ or ‘very good’ self-reported health in the former East and West German federal states by sex Source: SOEP 1994-2015 [14], own calculations

Findings on the development of subjective health are also made possible by examining Socio-Economic Panel data. There are only slight differences between the East and West German federal states in regard to the proportion of 18- to 79-year-old women and men who describe their overall health as either good or very good. These differences were slight even at the beginning of the 1990s. During the past 20 years, the proportion of women and men who estimated their health as good or very good has not changed substantially. This applies to both the East and the West German federal states, as is confirmed by a differentiated analysis for the 18- to 39 and 40- to 54-year old groups. The 55- to 79-year-old group is the only one that shows an increase in the proportion of women and men with either good or very good subjective health. For the West German federal states, this trend is much stronger than for the East German federal states. As a result, for men in this age group in particular, the differences between the East and the West are now more pronounced than at the beginning of the 1990s (Figure 7).

### 3.6 Smoking

**Figure 8:**
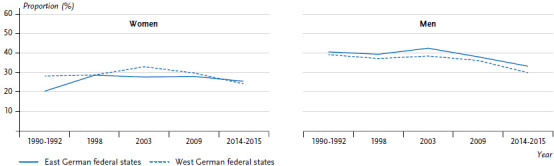
Prevalence trends in smoking in the 25- to 69-year-old population in the former East and West German federal states by sex Source: Health surveys of the Robert Koch Institute, updated according to Lampert 2010 [42]

The development of tobacco use in the 25- to 69-year-old population in East and West Germany between 1990 and 2015 can be traced using data from the health surveys of the Robert Koch Institute (Figure 8). Shortly after the reunification, the proportion of male smokers was at 40.6% in the East German federal states, slightly higher than the 39.2% recorded for the West German federal states [42]. Male tobacco use has been in decline since the 2000s, while the slight difference between East and West has remained more or less stable. Overall, fewer women smoke than men. At the beginning of the 1990s, 20.5% of women in the East were smokers, compared to a significantly higher 28.3% in the West German federal states. By the end of the 1990s, these figures for East and West German women had converged. Since the early 2000s, the percentage of female smokers in the East German federal states has remained relatively constant. In contrast, the number of female smokers in the West German federal states initially rose, before declining sharply after 2003. Most recently, the proportion of female smokers has even been slightly lower in the West than in the East (Figure 8).

The percentage of smokers over the age of 15 in individual federal states can be calculated using 2017 Microcensus data (Table 2) [15]. The highest rates are found among men from Mecklenburg-Western Pomerania (33.4%). Rates in Thuringia, Saxony-Anhalt, and the city-states Berlin and Bremen are also relatively high (around 30%). Men from Saarland, Hesse, the Rhineland Palatinate and Bavaria smoke less often (<25%). Rates for women are highest in Bremen (24.2%), followed by Mecklenburg-Western Pomerania (22.1%) and Thuringia (21.7%). In a comparison of federal states, smoking rates for women from Bavaria and Saxony are the lowest (16.6%).

### 3.7 Obesity

**Figure 9:**
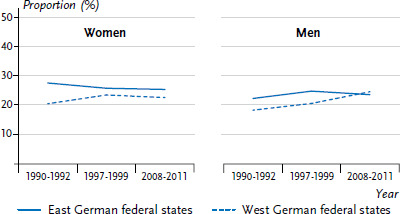
Prevalence trends in obesity in 25- to 69-year-olds in the former East and West German federal states by sex Source: Health surveys of the Robert Koch Institute, modified according to Finger et al. 2016 [9]

During the initial years after German reunification, measured data indicated a significantly higher prevalence of obesity for both genders in the former East compared to the former West. Since then, prevalences in Germany have continued to rise [43], while the differences between East and West German federal states have gradually decreased [9]. The most recent available data (DEGS1) suggests that, in regard to the distribution of obesity, there are no longer any significant differences between the East and West for 25- to 69-year-old men. Small differences persist for women, with a higher prevalence in the East (Figure 9) [7], although it should be noted that the DEGS1 data was collected between 2008 and 2011. The next data set will be provided by the Health and Nutrition Survey in Germany (gern survey), which the Robert Koch Institute will jointly conduct with the Max Rubner Institute between 2020 and 2022.

Self-reported data on height and weight from the Microcensus can be used to analyse regional spread patterns. When doing so, it is important to note that prevalences based on self-reported data are usually far lower than those calculated using measurement data, since a certain proportion of participants tends to underestimate their weight and overestimate their height [44]. According to 2017 Microcensus data, the highest prevalence (totalling over 20%) are found in Mecklenburg-Western Pomerania, Saxony-Anhalt and Thuringia. Obesity levels are lowest in the city-states of Hamburg and Berlin (Table 3). An interpretation of these findings needs to take into account the – on average – younger age of the population in the city-states [16].

### 3.8 Physical inactivity

**Figure 10:**
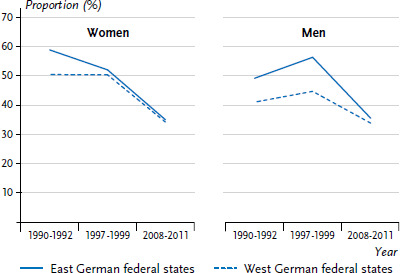
Prevalence trends in physical inactivity in 25- to 69-year-olds in the former East and West German federal states by sex Source: Health surveys of the Robert Koch Institute, modified according to Finger et al. 2016 [9]

Data from the Robert Koch Institute health examination survey shows that, shortly after German reunification, levels of physical activity were lower in the East German federal states than in the West (Figure 10). In the 25- to 69-year-old age group, 49.2% of men in the East and 41.0% in the West stated that they did not do any sport. The corresponding figures for women were 58.7% in the East and 50.3% in the West German federal states. Towards the end of the 1990s, the differences between East and West had increased for men, whereas for women there were no longer any detectable differences. Most recently, levels of physical inactivity have decreased significantly in both the East and the West German federal states. There are currently no differences between East and West either for women or for men.

**Figure 11:**
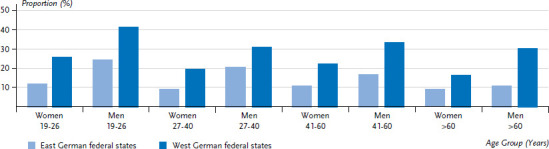
Sports club members in relation to respective total populations in the former East and West German federal states (as of 31 December 2017) by sex and age Source: German Olympics Sports Confederation 2018 [20]

The statistics of the German Olympics Sports Confederation are also informative [20]. They indicate that in 2018, a significantly smaller percentage of people in the East were members of a sports club than in the West German federal states. This is apparent across all age groups regardless of gender (Figure 11). The highest levels of sports club membership can be found in the Saarland (37%), in Rhineland Palatinate (35%), as well as in Bavaria, Baden-Wuerttemberg and Hesse (all around 34%). In comparison, only 13% to 18% of the East German population are members of a sports club.

### 3.9 Differences on a smaller scale

In many cases, making distinctions between East and West or individual federal states is inadequate when analysing regional health differences. It should be noted that there can be substantial regional differences even within federal states in regard to a number of health indicators. For this reason, regional analyses on a smaller scale than the federal state level should be conducted whenever possible, for example in Germany’s 96 planning regions (Raumordnungsregionen), 401 administrative rural and urban districts, and 4,504 collective municipalities.

**Figure 12:**
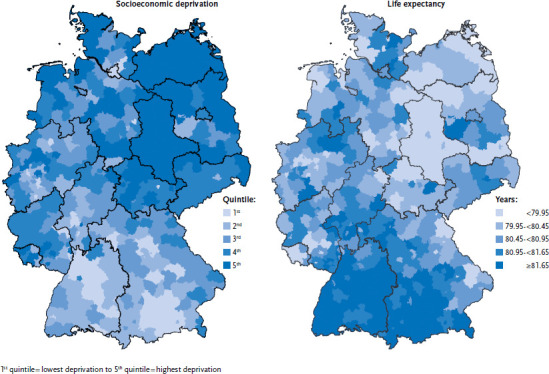
Regional differences (rural and urban districts) in relation to socioeconomic deprivation and average life expectancy at birth Source: Kroll et al. 2017 [22]; INKAR, own calculations

Figure 12 shows the differences between average life expectancy at birth and socioeconomic deprivation in rural and urban districts. In Saxony-Anhalt, Thuringia and Brandenburg, there is a relatively large number of rural and urban districts with a low average life expectancy, whereas in Bavaria and Baden-Wuerttemberg, there are many rural and urban districts where life expectancy is high. It is clear that socioeconomic deprivation is highest in rural and urban districts with a low average life expectancy, and vice versa. The difference in average life expectancy between rural and urban districts with the lowest and highest levels of socioeconomic deprivation is around five years.

For many other health indicators, too, a differentiated regional analysis demonstrably makes sense and can lead to different or additional findings. This can be seen in the case of sick days taken due to mental health issues. Data from company health insurance funds (Betriebskrankenkassen, BKK) members has shown that, in many rural and urban districts in Mecklenburg-Western Pomerania, Brandenburg and Saxony-Anhalt, the number of sick days due to mental health issues was far higher than the 2017 national average of 2.8 days. The same applies to some of the former West German federal states such as North Rhine-Westphalia, Lower Saxony and Saarland. On the other hand, the greatest deviation towards fewer sick days was found in Bavaria and Baden-Wuerttemberg, once again in rural and urban districts with a tendency toward lower levels of socioeconomic deprivation. However, there are also rural and urban districts in northern North Rhine-Westphalia, on Schleswig Holstein’s North Sea coast and in parts of Saxony that register significantly fewer sick days than the national average. Overall, these results demonstrate that a smaller-scale analysis is helpful, for example for regional planning in regard to healthcare structures, prevention, and the promotion of health [45].

## 4. Discussion

The results presented here show that shortly after German reunification, the health situation in the former East and West German federal states differed in many aspects. 30 years of common development has, in many cases, led to the convergence and in some cases to the equalisation of these differences. Remarkably, this equalisation was already taking place during the first 10 to 15 years after the reunification and can be ascribed to favourable developments that occurred faster in former East Germany than in former West Germany. Examples of such favourable developments include an increase in average life expectancy and a decrease in cardiovascular mortality. In some cases, equalisation actually occurred through negative trends, i.e. where the situation in the East was originally better but then became like the West. An example in this regard is the rise of tobacco use among women in former East Germany during the 1990s.

Data on the health situation of children and adolescents born after the reunification also indicate that the differences between the East and the West are no longer significant. The present article does not discuss these data; however, they were included in a report published by the Robert Koch Institute to commemorate the 20th anniversary of German reunification [8, 46]. According to this report, there have been no differences in the prevalence of allergic diseases and obesity amongst children and adolescents from the former East and West German federal states since the 2000s. There has also been a significant reduction in differences between East and West in regard to vaccination rates, which were initially higher in the former East German federal states, and in regard to participation rates for early detection examinations by children (U-Untersuchungen), which were more widely accepted in former West Germany. Based on these results, it can be assumed that differences between the East and the West will play an ever-diminishing role in future birth cohorts [8, 46].

The question of why the differences between East and West have diminished and why some regional disparities in health persist is not easy to answer. A significant contribution to the convergence of health opportunities in the East and West federal states was likely made by the increase in general living standards in former East Germany during the unification process, which was made possible by substantial investment and development aid. Contributing factors of note are economic restructuring, improvements in labour market conditions, improvements to urban development, housing conditions and environmental factors, and the rapid integration of the East into social security and healthcare systems.

Alongside these general processes, other developments likely played a role in diminishing (or maintaining) East-West differences. These can vary greatly depending on which health indicator is analysed. Any analysis of the continuing differences in sports club membership rates must allow for the fact that the role of club sport in mass sports was less important in the GDR than in former West Germany. The reason for this is that GDR sports clubs were more closely tied to promoting competitive sports. The higher child vaccination rates in the former East were probably also related to the compulsory GDR vaccination programme for many infectious diseases. Conversely, lower participation in early detection examinations in the former East can, to a certain degree, be explained by the fact that these programmes were first introduced in East Germany after the reunification.

In regard to health differences between federal states, and at the rural and urban district level, differences in living conditions and opportunities for social participation are particularly important. The present article has referred to these using the German Index of Socioeconomic Deprivation, and by referring to individual indicators such as the at-risk-of-poverty rate, unemployment rate or gross domestic product. To an extent, the correlations can be traced to the socio-spatial segregation of people in different life situations. However, the results of multi-level analyses indicate that independent of individual socioeconomic status, the quality of the social environment has an effect on people’s health prospects [22, 47].

The analysis of smaller-scale differences points to an increasing need, in the face of ever-diminishing differences between East and West, to apply regional criteria other than East and West or federal states. These could include smaller-scale approaches such as the NUTS2 regions, regional planning regions (Raumordnungsregionen) or district and municipality-based analytical approaches. As well as concepts orientated towards existing administrative boundaries, it could be useful to apply analysis that differentiates between urban and rural spaces, or, even more strongly differentiated, categorisations of district type or GPS-based health research.

To conclude, it is important to note that the development of both parts of Germany after the fall of the Berlin Wall did not take place in isolation but rather within the context of Europe. The breakdown of the socialist system in Europe caused similar upheavals in Poland, the Czech Republic, Hungary and other East European states. However, in comparison to East Germany, these countries did not become part of an economically stronger country and could not, therefore, count on associated investment and development aid. Against this historical backdrop, the extent and pace of the positive developments in health and life expectancy in former East Germany stand out [48, 49].

## Key statements

- As early as the 1990s there was a significant decrease in differences in life expectancy between former East and West Germany.
- In the last 30 years, mortality rates for cardiovascular disease have reached a similar level in the former East German federal states to those of the former West German federal states.
- Depression is slightly less common in the former East than in the former West.
- No differences in the prevalence of smoking or obesity persist between the former East and West.
- People in the former East are less likely to belong to a sports club than people in the former West.

## Figures and Tables

**Table 1:** Development of average life expectancy at birth (e_0_) and further life expectancy at age 65 (e_65_) in the former East and West German federal states by sex Source: Vital statistics [17]

	Women				Men			
	1991/1993	1999/2001	2007/2009	2015/2017	1991/1993	1999/2001	2007/2009	2015/2017
**e_0_**								
Germany	79.01	81.07	82.53	83.18	72.47	75.00	77.33	78.36
East German federal states	77.18	80.53	82.37	83.22	69.86	73.69	76.27	77.25
West German federal states	79.48	80.72	82.57	83.17	73.11	75.43	77.58	78.61
Difference	-2.30	-0.19	-0.20	0.05	-3.25	-1.74	-1.31	-1.36
**e_65_**								
Germany	18.02	19.44	20.52	21.00	14.34	15.79	17.22	17.80
East German federal states	16.69	18.90	20.20	21.05	13.26	15.17	16.75	17.44
West German federal states	18.35	19.59	20.60	20.99	14.58	15.92	17.32	17.89
Difference	-1.66	-0.69	-0.40	0.06	-1.32	-0.75	-0.57	-0.45

**Table 2:** Percentage of smokers aged over 15 by sex and federal state Source: Microcensus 2017 [15]

Federal state	Women %	Men %	Total %
Baden-Wuerttemberg	17.4	25.1	21.2
Bavaria	16.6	24.6	20.5
Berlin	21.3	29.9	25.5
Brandenburg	20.2	29.0	24.5
Bremen	24.2	30.9	27.4
Hamburg	18.9	27.8	23.2
Hesse	17.5	24.8	21.1
Mecklenburg-Western Pomerania	22.1	33.4	27.7
Lower Saxony	19.2	26.7	22.9
North Rhine-Westphalia	19.4	26.0	22.6
Rhineland-Palatinate	18.6	24.9	21.7
Saarland	17.7	23.5	20.6
Saxony	16.6	26.5	21.4
Saxony-Anhalt	20.0	29.8	24.8
Schleswig-Holstein	20.0	27.1	23.5
Thuringia	21.7	30.8	26.2
**Germany**	**18.6**	**26.4**	**22.4**

**Table 3:** Prevalence of obesity by sex and federal state Source: Microcensus 2017 [16]

Federal state	Women %	Men %	Total %
Baden-Wuerttemberg	13.2	16.4	14.9
Bavaria	12.9	17.3	15.2
Berlin	12.2	13.8	13.0
Brandenburg	17.1	19.5	18.3
Bremen	14.6	18.1	17.8
Hamburg	10.0	12.8	12.2
Hesse	14.0	18.2	16.1
Mecklenburg-Western Pomerania	20.0	23.5	21.8
Lower Saxony	14.5	18.5	16.6
North Rhine-Westphalia	14.6	18.4	16.5
Rhineland-Palatinate	15.3	19.9	17.7
Saarland	11.8	19.6	16.3
Saxony	17.8	18.2	18.0
Saxony-Anhalt	19.8	21.7	20.8
Schleswig-Holstein	12.8	17.9	15.4
Thuringia	19.5	21.4	20.4
**Germany**	**13.2**	**16.4**	**16.3**
